# Spatial variation in leaf nutrient traits of dominant desert riparian plant species in an arid inland river basin of China

**DOI:** 10.1002/ece3.4877

**Published:** 2019-01-13

**Authors:** Xiaolong Zhang, Jihua Zhou, Tianyu Guan, Wentao Cai, Lianhe Jiang, Liming Lai, Nannan Gao, Yuanrun Zheng

**Affiliations:** ^1^ Faculty of Environmental and Economics Shanxi University of Finance and Economics Taiyuan China; ^2^ University of Chinese Academy of Sciences Beijing China; ^3^ Key Laboratory of Plant Resources, West China Subalpine Botanical Garden, Institute of Botany Chinese Academy of Sciences Beijing China

**Keywords:** desert riparian ecosystems, global changes, groundwater, leaf nutrient traits, soil properties

## Abstract

Understanding how patterns of leaf nutrient traits respond to groundwater depth is crucial for modeling the nutrient cycling of desert riparian ecosystems and forecasting the responses of ecosystems to global changes. In this study, we measured leaf nutrients along a transect across a groundwater depth gradient in the downstream Heihe River to explore the response of leaf nutrient traits to groundwater depth and soil properties. We found that leaf nutrient traits of dominant species showed different responses to groundwater depth gradient. Leaf C, leaf N, leaf P, and leaf K decreased significantly with groundwater depth, whereas patterns of leaf C/N and leaf N/P followed quadratic relationships with groundwater depth. Meanwhile, leaf C/P did not vary significantly along the groundwater depth gradient. Variations in leaf nutrient traits were associated with soil properties (e.g., soil bulk density, soil pH). Groundwater depth and soil pH jointly regulated the variation of leaf nutrient traits; however, groundwater depth explained the variation of leaf nutrient traits better than did soil pH. At the local scale in the typical desert riparian ecosystem, the dominant species was characterized by low leaf C, leaf N, and leaf P, but high leaf N/P and leaf C/P, indicating that desert riparian plants might be more limited by P than N in the growing season. Our observations will help to reveal specific adaptation patterns in relation to the groundwater depth gradient for dominant desert riparian species, provide insights into adaptive trends of leaf nutrient traits, and add information relevant to understanding the adaptive strategies of desert riparian forest vegetation to moisture gradients.

## 1 INTRODUCTION

The desert riparian ecosystem, as a groundwater‐dependent ecosystem, protects the stability of the environment and provides critical ecosystem services to organisms in arid regions (Décamps et al., 2004; Ding et al., 2017; Stromberg, McCluney, Dixon, & Meixner, 2013). In arid inland river basins, particularly in the lower reaches, desert riparian forests composed mainly of *Populus euphratica* Oliv. an*d Tamarix* spp. are the core components of the riparian ecosystem, providing key habitats for many different species and functioning as a critical ecological defense against sandstorms and desertification (Ding et al., 2017; Zhu et al., 2016). In these groundwater‐dependent ecosystems, the groundwater dynamics are the key factor in the shaping and functioning of riverine ecosystems, and groundwater changes are likely the crucial determinant of the vegetation distribution of the desert riparian forest ecosystem (Tamea, Laio, Ridolfi, D′Odorico, & Rodriguez‐Iturbe, 2009). Due to global climate change and human disturbance, scarcity or variable distribution of water and nutrients make desert riparian forests highly sensitive to change, and alterations, particularly alterations in water condition, can have significant impacts on the desert riparian forest vegetation (Stromberg, Beauchamp, Dixon, Lite, & Paradzick, 2007; Tamea et al., 2009; Zhu et al., 2016).

Water availability is the major factor determining the structure and function of desert riparian ecosystems in arid inland river basins (Stromberg et al., 2013). Currently, the desert riparian vegetation–water relationship has been widely studied in eco‐hydrological research (Chen et al., 2008; Loheide & Gorelick, 2007; Stromberg et al., 2013). Plant leaves, as the main organs of photosynthesis, play a key role in determining plant survival and productivity in ecosystems, and their nutrient traits, including leaf carbon, leaf nitrogen, leaf phosphorus, and leaf potassium, are closely related to the structure and function of ecosystems (Güsewell, 2004; Wright et al., 2004). In arid regions, water conditions (e.g., precipitation) may alter the leaf nutrient traits, mainly through plant–soil feedback, and the leaf nutrient traits also change according to differences in species composition (Wang et al., 2015; Zhang et al., 2018a). Leaf decomposition and nutrient uptake and assimilation drive biogeochemical cycling to a certain degree, and these processes of resource investment and reinvestment are inherently survival strategies adapted to a chronic drought environment for plants in arid areas (Drenovsky & Richards, 2004; Simões, Calado, Madeira, & Gazarini, 2011). Therefore, quantification of the patterns and the main driving factors for leaf nutrient traits is critical for revealing the patterns of nutrient variation in arid environments and forecasting the response of desert riparian forests to the changes of water condition induced by global climate change.

Ecological stoichiometry, as an emerging comprehensive approach, yields new insights for researching the complex relationships of C/N/P stoichiometric traits at different ecosystem and spatial scales (Elser, Sterner, et al., 2000; Han, Fang, Guo, & Zhang, 2005; Reich & Oleksyn, 2004; Sterner & Elser, 2002). Elser, Fagan, et al. (2000) observed that plants survive in a relatively wide range of nutrient contents in terrestrial ecosystems, and the concentrations of C, N, P, and their stoichiometric ratios within plants to an extent reflect how a plant has adapted to the local habitat (Elser, Fagan, Kerkhoff, Swenson, & Enquist, 2010). Previous studies on arid and semiarid regions suggest that plant growth is mainly limited by the N element (Wang et al., 2015; Zhang et al., 2018a), but in other studies, P‐limited growth has also been detected (Zheng & Shangguan, 2007). These differences may be due to differences in nutrient limitations in different areas (Zhao et al., 2014). Currently, there has been increasing investigation on the patterns of leaf C/N/P stoichiometry and their relationships with climatic and geographical factors (Kerkhoff, Enquist, Elser, & Fagan, 2005; Mcgroddy, Daufresne, & Hedin, 2004; Reich & Oleksyn, 2004; Zhao et al., 2014). However, very few studies have addressed how the leaf nutrient traits of dominant riparian species respond to variations in groundwater depth, and in particular the relationship between groundwater depth and nutrient stoichiometry along a natural groundwater depth gradient in a hyperarid region.

The downstream of Heihe River in northwestern China is an ideal area for research on the response of plants to environment due to there are different natural desert riparian communities along groundwater depth gradient induced by river flow (Ding et al., 2017; Zhu et al., 2016). Groundwater provided by the river is considered the key factor in affecting vegetation growth and distribution (Chen et al., 2008; Tamea et al., 2009). However, exactly how groundwater depth and soil properties affect leaf nutrient traits along a moisture gradient remains largely unclear at the local scale in this hyperarid region, and further research is needed to reveal the mechanism of nutrient utilization and uptake and the distribution patterns of leaf nutrient traits of desert riparian vegetation, which will be valuable for restoring and managing desert riparian forest ecosystems in response to climate change.

In this study, 11 sites along a groundwater depth gradient were selected to examine responses of the leaf nutrient traits of dominant desert riparian species to groundwater depth in a desert riparian ecosystem. Our study was designed to (a) examine the patterns of leaf nutrient traits of dominant species along a natural groundwater depth gradient; (b) assess the relationship of leaf nutrient traits and soil physicochemical properties; (c) disentangle the main drivers affecting the variation of leaf nutrient traits. We hypothesized that groundwater depth play a key role on leaf nutrient traits, soil properties especially soil salinity and alkalinity may have important effect on leaf nutrient traits at specific distance from river.

## 2 MATERIALS AND METHODS

### 2.1 Study sites

The Heihe River is the second largest inland river in China, with a length of 821 km. The upper reaches are covered with alpine glaciers and permafrost, becoming the main source of regional runoff; runoff is primarily consumed in the middle reaches due to extensive deserts and farmland (Ding et al., 2017; Peng, Xiao, & Xiao, 2013). Over the past decades, anthropogenic grazing and farming in the middle reaches have led to the disappearance of most of the downstream surface runoff, lakes drying up, and groundwater depth increasing sharply, which has resulted in significant degradation of the desert riparian forest of *P. euphratica* (Zhu et al., 2016). A water conveyance project was implemented beginning in 2000; regular water conveyance has already caused groundwater levels to rise, and the riparian vegetation has correspondingly been restored (Ding et al., 2017; Fu, Chen, & Li, 2014).

**Figure 1:**
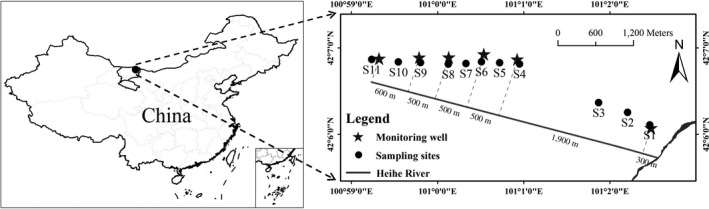
The locations of the sampling sites

Our study transect was established in a desert riparian forest ecosystem along a groundwater depth gradient in the downstream Heihe River, China (Figure 1). The region has a hyperarid desert climate, with an annual rainfall of approximately 37.4 mm, while the mean annual pan evaporation is 3,467.56 mm; more than 75% falls during the growing season (July to September), and the annual temperature is 8.57°C (Fu et al., 2014; Li, Yu, Li, & Zhao, 2016). The elevation changes relatively little from 921 m to 925 m. Groundwater provided by the Heihe River is the major water source available for maintaining local ecosystem stability (Li et al., 2016). The regional soil is gray‐brown desert soil. The vegetation types generally shift from riparian forest dominated by *P. euphratica* and *Tamarix ramosissima* Ledeb. to desert scrub dominated by* Reaumuria songarica* (Pall.) Maxim. along the river channel (Fu et al., 2014).

### 2.2 Experimental design and data collection

In early August 2015, eleven sampling sites were selected in a desert riparian ecosystem and sampled at the distances of 300, 800, 1,300, 2,200, 2,450, 2,700, 2,950, 3,200, 3,700, 4,000, and 4,500 m from Heihe River (Figure 1). Our field survey was conducted in one week. Due to the regular water conveyance project, floodwater only impacts the areas near the river bank, and the groundwater depth is relatively stable in the growing season (Ding et al., 2017; Fu et al., 2014). Groundwater depth data were obtained using a Hobo groundwater depth gauge from seven monitoring wells in 2010–2014 (http://westdc.westgis.ac.cn/), which located at 300, 2,200, 2,700, 3,200, 3,700, and 4,300 m from Heihe River. Apart from sites 1, 4, 6, 8, and 9, the groundwater depth data at other sites were obtained by cokriging interpolation method (Ahmadi & Sedghamiz, 2008; Hoeksema et al., 1989; Zhang et al., 2018b). At each site, three 5 m × 5 m plots were established randomly, where we collected leaf samples and soil samples (0–50 cm).

Along the groundwater depth gradient from S1 to S11, the dominant species were *T. ramosissima* (S1–S7, S9), *Karelinia caspica* (Pall.) Less. (S8), and *R. songarica* (S10, S11). At each site, fully expanded and healthy leaves were sampled from the dominant species in each plot, and then fresh weight of more than 100 g were gathered in envelopes and kept in an ice chest for further analysis. Leaf samples were grinded into a fine powder using a high speed mixer mill (MM400, Retsch, Haan, Germany) for nutrient analysis.

At each site, soil cores were collected every 10 cm at a soil depth of 50 cm, with three replicates. Soil moisture was determined gravimetrically via oven drying at 105°C for 72 hr. Soil bulk density was analyzed using a cutting ring (100 cm^3^ in volume) by the cutting ring method at 10, 20, 30, 40, 50 cm deep. Soil samples were air‐dried and then passed through a 2 mm sieve for chemical analysis.

Soil electrical conductivity and pH were analyzed through 1:5 and 1:1 soil:water suspensions using a portable electronic probes (Multiline F/SET‐3, WTW, Weilheim, Germany), soil available P and K content were determined according to the Olsen method and atomic absorption spectroscopy, respectively. Leaf C, Soil total C, leaf N, and soil total N were extracted by an elemental analyzer (Vario EL III, Elementar, Hanau, Germany). Leaf P and leaf K were analyzed by inductively coupled plasma optical emission spectrometer (iCAP 6300, Thermo Scientific, Waltham, USA).

### 2.3 Statistical tests

All species importance values determined the dominant species in each plant community and were obtained from the following formula (Curtis & Mcintosh, 1951):

(1) IV=(RC+RD+RF)/3

where IV is the importance value of the species, RC is the relative coverage, RD is the relative density, RF is the relative frequency.

The list of species composition and importance values in the studied area are shown in Supporting Information Table S1. At the each site, groundwater depth and leaf nutrient traits were presented as the mean ± *SD* (Supporting Information Table S2). The gravimetric analysis of soil moisture was expressed as the mean value for the layers of 0–30 cm and 30–50 cm; other physicochemical properties were expressed as the mean values for the layers at 0–50 cm soil depth.

Pearson's correlations were performed to determine the relationships between leaf nutrient traits and soil properties according to the level of significance. A generalized linear model with sampling location (distance from river) as a random effect was used to examine the relative importance of soil variables for the leaf nutrient traits, and one‐way ANOVA was used to explore the differences in leaf nutrient traits among different sampling sites, Tukey's test was used to determine statistically significant differences; homogeneity of variance and normality for all leaf nutrient traits data were assessed and calculated from the log10 transformation. Appropriate regression analysis was used to examine the relationships between leaf nutrient traits and groundwater depth according to high *R* ^2^ values and the level of significance. Significant differences were evaluated at *p* < 0.05, and all statistical analyses were performed using SPSS 22.0.

A redundancy analysis (RDA) was applied to determine the key environmental factors of the variation of leaf nutrient traits using soil properties and groundwater depth data. First, the marginal and conditional effects of environmental variables were obtained from forward selection in the redundancy analysis. Statistical testing for added environmental explanatory variables was performed with Monte Carlo tests (9,999 permutations) (Ter Braak & Smilauer, 2012). Compared to Marginal effects, conditional effects represented the effects of the explanatory variables on the leaf nutrient traits after the previous variable was eliminated through forward selection (Ding et al., 2017; Ter Braak & Smilauer, 2012). After the RDA, the key variables (*p* < 0.05) were included in the group of the key environment variables. Related to our hypotheses, variation partitioning model (VPM) was used to decompose the two groups of significant variables: groundwater depth and soil properties, and quantify the interpretation rate of the variation of leaf nutrient traits by separating the significant variables into the unique effects of each variable and the joint effects between variables (Heikkinen, Luoto, Kuussaari, & Pöyry, 2005). The redundancy analysis, variation partitioning, and forward selection were performed using CANOCO 5.0 for Windows (Ter Braak & Smilauer, 2012).

## 3 RESULTS

### 3.1 Leaf nutrient trait changes in response to groundwater depth

In all plant species, the average leaf C, leaf N, leaf P, and leaf K were 327.29, 13.88, 0.58, and 6.71 mg/g, respectively, and the leaf C/N, leaf N/P, and leaf C/P ratios were 24.41, 26.12, and 614.94, respectively (Supporting Information Table S2).

The ANOVA results showed significant differences for leaf C (*F* = 12.437, *p* < 0.001) among the different sampling sites. Leaf C ranged from 173.71 to 381.34 mg/g for different sampling sites (Supporting Information Table S2). Sites along the groundwater depth gradient differed significantly in parameters including leaf N, leaf P, leaf C/N, leaf N/P, leaf C/P. Leaf N ranged from 9.89 to 19.50 mg/g, leaf P ranged from 0.30 to 0.98 mg/g, leaf C/N ranged from 13.49 to 34.45, leaf N/P ranged from 13.22 to 34.73, leaf C/P ranged from 363.18 to 1,118.22, and leaf K ranged from 5.33 to 9.19 mg/g. Among leaf nutrient traits, leaf P had the largest variation coefficient (0.34) (Supporting Information Table S2).

**Figure 2:**
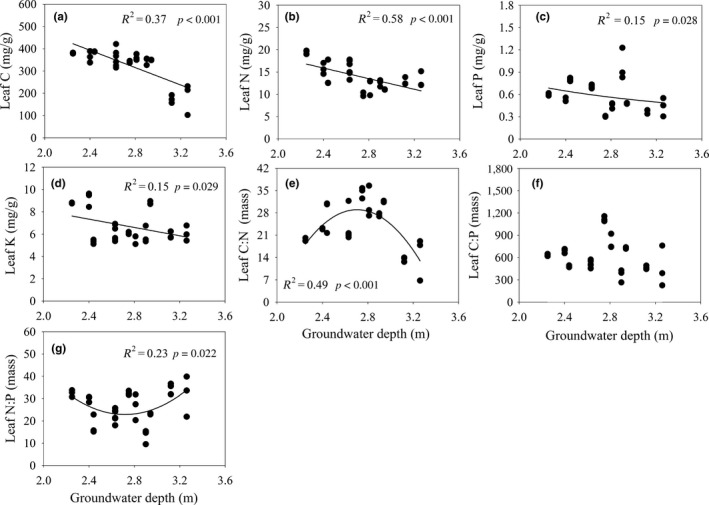
The variation of leaf nutrient traits with the groundwater depth gradient

Leaf C, leaf N, leaf P, and leaf K and C/N/P ratios varied with groundwater depth (Figure 2). Regression analysis showed that leaf C, leaf N, leaf P, and Leaf K significantly decreased with increasing groundwater depth (Figure 2a–d). Leaf C/N formed a unimodal pattern, increasing then decreasing sharply, and followed quadratic curves corresponding with groundwater depth (Figure 2e). Leaf C/P did not significantly change with increased groundwater depth (Figure 2f). In addition, leaf N/P showed an inverse trend to the pattern of leaf C/N with increasing groundwater depth (Figure 2g).

### 3.2 Leaf nutrient traits changes to soil properties

Leaf C, Leaf N, and leaf K had positive correlations with soil moisture in the 0–30 and 30–50 cm. Leaf C and leaf N were positively related to soil total carbon and soil total nitrogen. Leaf P was positively related to soil available phosphorus. Leaf N and leaf K were positively related to soil pH, whereas leaf P were negatively related to soil pH (Table 1).

**Table 1:** Pearson's correlation coefficients (R) among leaf nutrient traits and soil properties

	Leaf C	Leaf N	Leaf P	Leaf K	Leaf C:N	Leaf C:P	Leaf N:P
Soil moisture in 0–30 cm	0.491^**^	0.544^**^	0.208	0.455^**^	0.019	0.070	0.046
Soil moisture in 30–50 cm	0.500^**^	0.471^**^	0.246	0.419^*^	0.092	0.095	−0.010
Soil bulk density	−0.687^**^	−0.217	−0.373^*^	−0.168	−0.423^*^	−0.138	0.340
Soil total carbon (C)	0.526^**^	0.510^**^	0.287	0.486^**^	0.066	0.079	−0.006
Soil total nitrogen (N)	0.620^**^	0.524^**^	0.390^*^	0.288	0.127	−0.017	−0.188
Soil available phosphorus (P)	0.413^*^	0.158	0.429^*^	−0.310	0.194	−0.181	−0.424^*^
Soil available potassium (K)	0.582^**^	0.229	0.427^*^	0.102	0.314	0.106	−0.305
Soil C/N	−0.594^**^	−0.237	−0.269	−0.072	−0.331	−0.073	0.332
Soil electrical conductivity	0.404^*^	−0.178	0.328	−0.346^*^	0.462^**^	0.135	−0.423^*^
Soil pH	−0.279	0.428^*^	−0.376^*^	0.552^*^	−0.533^**^	0.014	0.634^**^

^*^ *p* < 0.05. ^**^ *p* < 0.01.

The results of generalized linear model showed that soil bulk density, soil total C and soil C/N had significant effects on leaf C, these variables could explain 19.7%, 22.5%, and 36.1% of the variation of leaf C, respectively. Soil pH had significant effect on leaf C:N and could explain 46.6% of the variation of Leaf C:N (Table 2).

**Table 2:** Effects of soil properties on the variations in leaf C, leaf N, leaf P, leaf K, and leaf C/N/P ratios

	*df*	Leaf C		Leaf N		Leaf P		Leaf K		Leaf C/N		Leaf C/P		Leaf N/P	
		SS%	*p*	SS%	*p*	SS%	*p*	SS%	*p*	SS%	*p*	SS%	*p*	SS%	*p*
Soil moisture (0–30 cm)	1	1.0	0.623	8.6	0.310	9.0	0.261	5.5	0.383	0.9	0.747	0.1	0.944	0.0	0.996
Soil moisture (30–50 cm)	1	1.3	0.569	3.6	0.507	8.5	0.274	0.0	0.964	0.4	0.832	0.1	0.941	0.9	0.789
Soil bulk density	1	19.7	0.042*	1.7	0.648	4.1	0.444	0.7	0.755	15.5	0.184	0.0	0.976	12.9	0.329
Soil total nitrogen	1	6.8	0.199	0.2	0.861	0.6	0.772	4.5	0.430	0.0	0.961	0.0	0.985	0.3	0.888
Soil total carbon	1	22.5	0.032*	10.8	0.257	3.6	0.475	1.7	0.621	0.6	0.796	0.5	0.857	1.7	0.719
Soil C/N	1	36.1	0.009**	3.9	0.490	10.6	0.223	10.3	0.235	1.5	0.670	0.2	0.912	0.3	0.884
Soil available P	1	0.1	0.866	3.3	0.527	0.5	0.780	1.7	0.629	7.5	0.353	5.2	0.571	0.2	0.893
Soil available K	1	1.3	0.570	8.4	0.317	1.5	0.638	7.3	0.321	1.4	0.690	0.0	0.981	3.0	0.632
Soil pH	1	6.6	0.204	31.4	0.061	10.5	0.226	0.9	0.721	46.6	0.028*	2.3	0.708	48.2	0.067
Soil electrical conductivity	1	0.2	0.806	1.5	0.672	5.5	0.378	16.9	0.135	0.0	0.974	4.4	0.604	2.4	0.669

^*^ *p* < 0.05. ^**^ *p* < 0.01.

### 3.3 Analysis of the controlling factor of leaf nutrient traits

**Figure 3:**
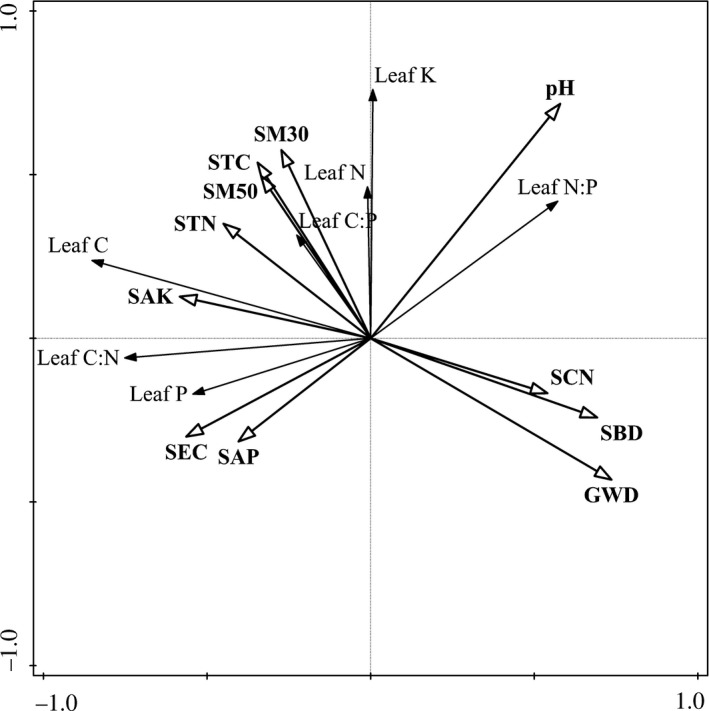
Redundancy analysis (RDA) ordination of all variables for leaf nutrient traits. Abbreviations: groundwater depth (GWD), soil moisture in 0–30 cm (SM30), soil moisture in 30–50 cm (SM50), soil bulk density (SBD), soil total carbon (STC), soil total nitrogen (STN), soil C:N (SCN), soil available P (SAP), soil available K (SAK), soil electrical conductivity (SEC)

The Monte Carlo test with forward selection in RDA for the variation of leaf nutrient traits showed that groundwater depth and soil pH had significant effects (*p* < 0.05) (Table 3). The RDA ordination also showed that the first axis represent the variation of groundwater depth, while the second axis represent the variation of soil pH (Figure 3).

**Table 3:** Results of marginal and conditional effects calculated from the forward selection with Monte Carlo test in a redundancy analysis using both groundwater depth and soil properties data

Marginal effects		Conditional effects		*p*	*F*
Variables	Percentage	Variables	Percentage		
Groundwater depth	21.2	Groundwater depth	21.2	0.001	8.3
Soil pH	17.7	Soil pH	17.9	0.001	8.8
Soil bulk density	17.3	Soil available K	3.9	0.124	2.0
Soil available K	12.2	Soil C/N	1.7	0.443	0.8
Soil electrical conductivity	12.0	Soil bulk density	1.6	0.454	0.8
Soil C/N	10.5	Soil total carbon	1.5	0.464	0.8
Soil total nitrogen	10.4	Soil total nitrogen	1.3	0.527	0.6
Soil total carbon	8.7	Soil moisture in 0–30 cm	0.9	0.676	0.4
Soil available P	8.3	Soil electrical conductivity	0.9	0.707	0.4
Soil moisture in 0–30 cm	7.6	Soil available P	0.6	0.798	0.3
Soil moisture in 30–50 cm	7.5	Soil moisture in 30–50 cm	0.1	0.990	<0.1

The variation partitioning model indicated that groundwater depth, soil pH, and their interaction explained 35% of the variation. The independent effect of groundwater depth had a large contribution, accounting for 20% of the variation in leaf nutrient traits, and the independent effect of soil pH had a small contribution, accounting for 16.4% of the variation in leaf nutrient traits.

## 4 DISCUSSION

### 4.1 Leaf nutrient traits in desert riparian ecosystem

In the lower reaches of Heihe River, our result showed that the average leaf C of the dominant species was 327.29 mg/g (Supporting Information Table S2), and lower than the mean values of global flora and of Loess Plateau (Elser, Fagan, et al., 2000; Zheng & Shangguan, 2007). Leaf N and leaf P were lower than that in the Gobi desert region of the Heihe River, and lower than the mean values of global flora and of other arid and semiarid regions (Reich & Oleksyn, 2004; Wang et al., 2015; Zhang et al., 2018a; Zheng & Shangguan, 2007). However, leaf C/P and leaf N/P were higher than that in other regions (Han et al., 2005; Reich & Oleksyn, 2004; Wang et al., 2015; Zheng & Shangguan, 2007). It is suggested that the dominant species was characterized by lower leaf C, leaf N, and leaf P, but higher leaf N/P and leaf C/P. In this study, the lower values of leaf C might be due to drought and salt stress. In this hyperarid region, plants need to reduce their water potential and stomatal conductance to minimize drought and salt stress during the growing season. In this case, photosynthesis may have been restricted, and the salt stress (high soil electrical conductivity) increasing the metabolic costs may have led to decreased C fixation (Chaves, Flexas, & Pinheiro, 2009; Dodd & Donovan, 1999; McCree, 1986). Leaf C dropped rapidly when groundwater depth was higher than 3 m (Figure 2), because *R. songarica* community suffered serious water stress in this hyperarid region (Fu et al., 2014), thus C fixation deceased, and species difference possibly was another reason, further research is needed in controlled conditions in future. The lower mean values of leaf N and leaf P along the groundwater depth gradient appeared to be attributable mainly to the dominant species *(T. ramosissima*). Because *T. ramosissima* is in flowering and fruiting stages from July to September, it is undergoing storage and the transfer of genetic material to reproductive organs (Rong, Liu, Xia, Lu, & Guo, 2012); therefore, leaf N and leaf P along the groundwater depth gradient were relatively low. Our results were based on samples taken during the growing season, and leaf N and leaf P may vary seasonally; thus, long‐term (e.g., in different seasons or years) field investigation is necessary in the future.

In this study, the high values of leaf C/P might also be attributable mainly to the dominant species (*T. ramosissima* and *R. songarica*), which had a slow growth rate and low photosynthetic rate due to drought (annual precipitation 30–40 mm) and salt stress (high soil electrical conductivity), thereby leading to greater carbon allocation compared with nitrogen and phosphorus allocation to leaves. Our results showed that leaf C/N and leaf C/P in desert riparian region were significantly higher than that in Gobi desert region indicating that the Gobi desert plants grew more rapidly in the growing season due to the limited rainfall; however, the desert riparian vegetation rather defense against habitat stress and differed from the Gobi desert vegetation's growth adaptation strategy in the growing season.

In arid terrestrial ecosystems, scarce precipitation causes poor soil leaching, low loss of P from soil weathering, and a low biodiversity level and vegetation cover, leading to soil P content being relatively abundant compared to soil N content, which could explain why nitrogen is more likely to be the key limiting factor in desert ecosystems (Vitousek & Howarth, 1991; Vitousek, Porder, Houlton, & Chadwick, 2010). These results were also confirmed by our results (Zhang et al., 2018a) and those of other studies (Vitousek et al., 2010; Wang et al., 2015; Wang, Yang, & Ma, 2008). Previous studies on the different ecosystems suggest that leaf N/P < 14 demonstrates N limitation, leaf N/P > 16 demonstrates P limitation, and 14 < leaf N/P < 16 indicates either N or P limitation, or both (Aerts & Chapin, 2000; Koerselman & Meuleman, 1996). Our result showed that the average leaf N/P was 26.12, indicating that dominant desert riparian vegetation was largely constrained by P. This result differs from previous studies in other desert ecosystems (Vitousek & Howarth, 1991; Wang et al., 2015; Zhang et al., 2018a) while is consistent with the results from the Loess Plateau study in China (Zheng & Shangguan, 2007). Some research indicated that P is the main growth‐limiting nutrient of plant communities because of low soil phosphorus levels in China (Han et al., 2005). In this hyperarid region, this difference apparently was in response to a combination of factors: first, the dominant species of the community (*T. ramosissima*) is a typical shrub associated with endophytic diazotrophic bacteria, resulting in an increase in N fixation by plants (Xu, Luo, Wang, & Wang, 2014). Second, soil salt stress affects the absorption of P because many Cl^−^, SO_4_^2−^ and other anions exist in the soil and compete with P, resulting in a decrease in P uptake by plants (Balba, 1995), thereby resulting in a high N/P ratio. Moreover, to survive under the stress of extremely arid environments with high temperature, plants tend to have lower growth rates, which might result in a higher N/P ratio. In fact, some research indicated that the leaf N/P stoichiometric ratio was impacted by plant genetic characteristics and survival strategies and was closely associated with habitat complexity (Aerts & Chapin, 2000; Kerkhoff et al., 2005; Koerselman & Meuleman, 1996). Due to the low plant diversity in arid regions, so the leaf N/P of dominant species can be indicative of nutrient limitation of the desert riparian plant community, to some extent. Güsewell and Koerselman (2002) reported that the leaf N/P accurately determined the nutrient limitation of plant growth at the community level. Therefore, further detailed field investigations in different seasons and artificial controlled experiment should be performed to reveal the nutrient limitation patterns of different plant functional groups or at the community level.

### 4.2 Variations of leaf nutrient traits and its influencing factors

Leaf trait–environment relationships are important to explain and predict the underlying mechanisms of the pattern of leaf nutrient traits along environmental gradients and to identify ecosystem nutrient limitations (Güsewell, 2004; Kerkhoff et al., 2005; Reich & Oleksyn, 2004; Zhao et al., 2014). In the Gobi desert region of Heihe River, the patterns of leaf nutrient traits controlled by precipitation were not significant, but soil moisture was better able to explain the variations in leaf nutrient traits (Zhang et al., 2018a). These results indicated that water conditions might be the main driving factor affecting the variations of leaf nutrient traits in arid regions. Our results showed that groundwater depth and soil pH jointly influenced the pattern of leaf nutrient traits in the desert riparian vegetation (Table 3). Apart from groundwater depth, soil properties especially saline‐alkali properties may also have had important effect on leaf nutrient traits at local scale in this hyperarid region. In addition, the interactions of groundwater depth and soil pH negatively contributed to the explained variation in leaf nutrient traits; however, the independent effects of groundwater depth and soil pH were positively contributed to explaining this variation. The negative contribution of the interaction indicated that the majority of the relationships between the two factors (groundwater depth and soil pH) are likely suppressive rather than additive (Heikkinen et al., 2005).

Leaf nutrient traits showed a significant groundwater depth gradient in the desert riparian ecosystem of the Heihe River (Figure 2). Groundwater was the critical water resource for the survival of desert plants, and it also regulated plant growth and nutrient fluxes through interactions with environmental factors (Tamea et al., 2009; Zhang et al., 2018b). In our previous study, soil nutrients decreased significantly along the groundwater depth gradient (Zhang et al., 2018b), and leaf nutrient traits were closely associated with soil properties (Table 1); increased groundwater depth and soil nutrient properties affected the leaf C, leaf N, leaf P, and leaf K, as these factors all decreased significantly with groundwater depth. In the desert riparian, vegetation with shallow groundwater, water conditions, and soil nutrients at these sites might be generally sufficient, and more nutrients could be used by plants. With increasing groundwater depth, the depletion of soil nutrients from low nutrient availability in the soil resulted in a decrease of nutrient uptake by plants. In this hyperarid region, soil nutrients might be the main factor affecting the leaf nutrient traits at the local scale; however, the soil nutrient content is regulated by groundwater, and this could explain why groundwater depth was the largest contributor to the leaf nutrient traits in the variation partitioning model. Meanwhile, soil pH was better able to explain the variations in leaf nutrient traits, in our results and together with those from previous studies, suggests that soil saline‐alkali imposes a common limitation on plant growth and distribution patterns, largely due to the soil parent material in arid regions (Liu, Wang, & Xi, 2008; Zhou, Chen, & Li, 2010).

## CONFLICTS OF INTEREST

None declared.

## AUTHOR CONTRIBUTIONS

YRZ conceived and designed the experiment; TYG and XLZ performed materials and data analysis; XLZ wrote the paper; LHJ, LML, NNG, JHZ, XLZ, WTC, and TYG carried out field investigations.

## Data Availability

Leaf nutrient traits and soil properties contents data have been deposited in the Dryad Digital Repository. https://doi.org/10.5061/dryad.rg25909.
