# Supplementary material for: Spatial variation in leaf nutrient traits of dominant desert riparian plant species in an arid inland river basin of China
Source: Ecol Evol. 2019 Jan 13;9(3):1523–31. doi: 10.1002/ece3.4877 (PMC6374681; doi:10.1002/ece3.4877)
Supplement: Supplementary file 1 [file ECE3-9-1523-s001.doc]

***Table S1*** *Species composition and importance value of sampling sites. Site code is same as Figure 1. Shrub layer: TR, Tamarix ramosissima; LR, Lycium ruthenicum; RS, Reaumuria songarica; CM, Calligonum mongolicum; Herb layer: SA, Sophora alopecuroides; KC, Karelinia caspia; PH, Peganum harmala; SL, Salsola laricifolia; TM, Taraxacum mongolicum; BD, Bassia dasyphylla; OL, Olgaea lomonossowii; AS, Agriophyllum squarrosum; LT, Lactuca tatarica*

| Sites | Shrub layer | | | | Herb layer | | | | | | | | |
| --- | --- | --- | --- | --- | --- | --- | --- | --- | --- | --- | --- | --- | --- |
|  | TR | LR | RS | CM | SA | KC | PH | SL | TM | BD | OL | AS | LT |
| S1 | 0.7 |  |  |  | 0.12 |  | 0.05 | 0.11 | 0.02 |  |  |  |  |
| S2 | 0.7 |  |  |  | 0.16 |  |  | 0.08 |  |  | 0.04 |  | 0.02 |
| S3 | 0.51 | 0.19 |  |  |  | 0.20 | 0.07 |  |  | 0.01 |  | 0.02 |  |
| S4 | 0.96 | 0.04 |  |  |  |  |  |  |  |  |  |  |  |
| S5 | 1 |  |  |  |  |  |  |  |  |  |  |  |  |
| S6 | 1 |  |  |  |  |  |  |  |  |  |  |  |  |
| S7 | 0.71 | 0.09 |  |  |  | 0.20 |  |  |  |  |  |  |  |
| S8 |  |  |  |  |  | 1 |  |  |  |  |  |  |  |
| S9 | 0.68 | 0.12 |  |  |  | 0.2 |  |  |  |  |  |  |  |
| S10 |  |  | 0.87 | 0.13 |  |  |  |  |  |  |  |  |  |
| S11 |  |  | 1 |  |  |  |  |  |  |  |  |  |  |

**Table S2** Leaf nutrient traits of dominant species and mean groundwater depth along the downstream Heihe River. Values (Means ± standard deviation). Site code is same as Figure 1.

| Site | Distance from River | Groundwater Depth (m) | C  (mg g^-1^) | N  (mg g^-1^) | P  (mg g^-1^) | K  (mg g^-1^) | C/N (mass) | C/P  (mass) | N/P (mass) |
| --- | --- | --- | --- | --- | --- | --- | --- | --- | --- |
| S1  *Tamarix ramosissima* community | 300 m | 2.25± 0.14 | 380.18  ± 2.12 | 19.50  ± 0.42 | 0.60  ± 0.02 | 8.80  ± 0.04 | 19.50  ± 0.52 | 631.61  ± 15.17 | 32.41  ± 1.51 |
| S2  *Tamarix ramosissima* community | 800 m | 2.40 ± 0.18 | 363.52  ± 25.55 | 15.76  ± 1.23 | 0.53  ± 0.03 | 9.19  ± 0.65 | 23.08  ± 0.22 | 690.14  ± 28.84 | 29.90  ± 1.28 |
| S3  *Tamarix ramosissima* community | 1300 m | 2.44 ± 0.17 | 386.49  ± 1.25 | 14.30  ± 3.02 | 0.80  ± 0.02 | 5.33  ± 0.19 | 27.77  ± 5.27 | 483.35  ± 12.25 | 17.94  ± 4.27 |
| S4  *Tamarix ramosissima* community | 2200 m | 2.63 ± 0.03 | 381.34  ± 40.05 | 15.95  ± 2.38 | 0.70  ± 0.03 | 6.76  ± 0.23 | 24.51  ± 6.30 | 542.43  ± 37.42 | 22.82  ± 4.15 |
| S5  *Tamarix ramosissima* community | 2450 m | 2.63 ± 0.08 | 335.66  ± 28.04 | 15.78  ± 1.53 | 0.71  ± 0.02 | 5.53  ± 0.14 | 21.29  ± 0.34 | 473.31  ± 29.93 | 22.25  ± 1.73 |
| S6  *Tamarix ramosissima* community | 2700 m | 2.81 ± 0.17 | 361.18  ± 13.62 | 11.91  ± 1.84 | 0.45  ± 0.04 | 5.57  ± 0.40 | 30.84  ± 5.01 | 803.02  ± 101.5 | 26.57  ± 5.83 |
| S7  *Tamarix ramosissima* community | 2950 m | 2.75 ± 0.19 | 340.31  ± 4.67 | 9.89  ± 0.46 | 0.30  ± 0.01 | 6.07  ± 0.11 | 34.45  ± 1.71 | 1118.22  ± 34.3 | 32.49  ± 0.93 |
| S8  *Karelinia caspia* community | 3200 m | 2.90 ± 0.34 | 345.37  ± 16.23 | 12.56  ± 0.75 | 0.98  ± 0.21 | 5.88  ± 0.77 | 27.52  ± 0.53 | 363.18  ± 85.77 | 13.22  ± 3.20 |
| S9  *Tamarix ramosissima* community | 3700 m | 2.94 ± 0.23 | 349.36  ± 1.02 | 11.08  ± 0.06 | 0.48  ± 0.01 | 8.80 ±0.14 | 31.52  ± 0.25 | 731.70  ± 11.72 | 23.21  ± 0.29 |
| S10  *Reaumuria songarica* community | 4000 m | 3.12 ± 0.12 | 173.71  ± 17.49 | 12.86  ± 0.86 | 0.37  ± 0.03 | 5.89  ± 0.32 | 13.49  ± 0.73 | 467.71  ± 23.65 | 34.73  ± 2.46 |
| S11  *Reaumuria songarica* community | 4500 m | 3.26 ± 0.24 | 183.08  ± 70.18 | 13.12  ± 1.78 | 0.44  ± 0.12 | 6.06  ± 0.68 | 14.57  ± 6.79 | 459.69  ± 273.44 | 31.79  ± 9.12 |
| Mean | – | – | 327.29  ± 75.58 | 13.88  ± 2.72 | 0.58  ± 0.20 | 6.71  ± 1.47 | 24.41  ± 6.82 | 614.94  ± 214.48 | 26.12  ± 6.85 |
| CV | – | – | 0.23 | 0.20 | 0.34 | 0.22 | 0.28 | 0.35 | 0.26 |
